# Transparent Memory Tests Based on the Double Address Sequences

**DOI:** 10.3390/e23070894

**Published:** 2021-07-14

**Authors:** Ireneusz Mrozek, Vyacheslav N. Yarmolik

**Affiliations:** 1 Faculty of Computer Science, Bialystok University of Technology, 15-351 Białystok, Poland; 2 Faculty of Computer Science, Belarusian State University of Informatics and Radioelectronics, 220013 Minsk, Belarus; yarmolik@bsuir.by

**Keywords:** memory testing, RAM, transparent test, march test

## Abstract

An important achievement in the functional diagnostics of memory devices is the development and application of so-called transparent testing methods. This is especially important for modern computer systems, such as embedded systems, systems and networks on chips, on-board computer applications, network servers, and automated control systems that require periodic testing of their components. This article analyzes the effectiveness of existing transparent tests based on the use of the properties of data stored in the memory, such as changing data and their symmetry. As a new approach for constructing transparent tests, we propose to use modified address sequences with duplicate addresses to reduce the time complexity of tests and increase their diagnostic abilities.

## 1. Introduction

Semiconductor memory is a crucial part of today’s electronic systems. The percentage of silicon areas devoted to memory components in systems-on-chip (SoCs) is still on the rise [1]. Modern computers typically contain a variety of embedded memory arrays, such as caches, branch prediction tables or priority queues for instruction execution, along with random access memory (RAM). Fault-free memory operations are crucial for the correct behavior of the complete embedded system.

Moreover, memory chips are very often designed to exploit the technology’s limits (to get the highest storage density and access speed), which makes them prone to defects. Hence, efficient techniques for production testing and for periodic maintenance testing are mandatory to guarantee the required quality standards. However, advances in memory technology and system design have turned memory testing into a nontrivial task [2,3,4]. The complexity of the memory chips makes fault modeling and testing an evermore challenging problem. As a result, testing semiconductor memories is becoming a major cost factor in the production of memory chips for modern computers. Therefore, the selection of the most appropriate diagnostic techniques, test algorithms and target set of fault models is still a very hot topic in both academia and industry.

When testing the memory devices (MD) of modern computing systems, one of the main criteria, in addition to effective fault detection, is the ability of tests to restore operational data after each test session [5]. This job can be realized by transparent tests [6,7,8,9].

The transparent technique is a well-known memory testing approach that retrieves the initial contents of the memory once the test phase has been finished. It eliminates the problem of restoring the RAM contents after the system function has been interrupted for a periodic memory testing procedure. It is therefore suitable for periodic field testing while allowing for preserving the memory content. A transparent BIST (built-in self-test) is based on a transparent march test that uses the initial memory data to derive the test patterns. The write data can be either the read value or its opposite value. A transparent test algorithm ensures that the last write data are always equal to the first values read in order to satisfy the transparency property. The basic principle is that during testing, the memory stored data are complemented an even number of times. Several transparent test solutions can be found in [7,8,10,11]. However, the traditional approaches for transforming nontransparent march algorithms to transparent tests imply substantial amounts of test time needed for calculating the fault-free signatures. The prediction phase for fault-free signature calculation takes up to 50% of the transformed march test complexity. To avoid this overhead, adaptive signature analyses [11] and symmetric memory testing [12] have been proposed. In both cases, the final results of the test procedure rely on the only one comparison of the fault-free signature with the real signature obtained during the test session, which reduces the ability to detect and locate the memory faults.

In this paper, we propose the use of modified address sequences to build a new class of transparent test of memory and improve the efficiency of transparent testing.

## 2. Transparent Tests

The term transparent has been used to define the main property of transparent tests, which is the preservation of data stored in memory after the testing procedure.

### 2.1. Introduction to Transparent Tests

The first systems for transparent periodic testing of memory devices used backup memory modules for temporary data storage. This approach requires memory duplication, which is time consuming for data transfer, and is not commonly used in modern applications due to the steady growth in the volume of their memory devices. Due to these limitations, a need arose for new solutions for the implementation of transparent memory testing.

One of the first ideas based on a radical new approach was presented by B. Koneman at the Design for Testability seminar in 1986. The proposed technology used signature analysis with the property of linearity, which led to masking of some faults [7,13]. The test procedure consisted of a small number of steps, and its effectiveness depended on the masks determining the validity of testing. However, most of the fault models used to describe real memory defects are quite complicated, and their detection requires the use of more complex testing procedures.

Due to the linear complexity, regularity, symmetry and simplicity of the hardware implementations, the march tests are usually a preferred method, and often the only reasonable method, for RAM testing [14,15,16,17,18,19]. Therefore, to maximize the efficiency of transparent testing, a method based on the use of classic march tests was proposed by M. Nicolaidis [7,8]. It has been shown that an arbitrary march test can be converted to its transparent version and provide almost the same fault-detecting ability as the original test. Due to the changing contents of the memory between testing procedures, transparent tests guaranteed, in principle, the detection of all kinds of memory faults after repeated use [13,20]. However, the implementation of such transparent tests requires a significant increase in their time complexity, reaching 40–50% for most known march tests. In addition, this technology does not guarantee 100% coverage, even for single faults, due to the masking effect [9,21]. Another drawback of the technology based on classical transparent testing is associated with a decrease in diagnostic ability, which is caused by the difficulty of obtaining information about the type and location of the memory faults.

A further development in transparent testing was the emergence of symmetric transparent tests [7,9,22]. These tests utilize the symmetry of the data read from the memory during their testing to avoid the time-consuming phase of obtaining the reference signature, and accordingly, reduce the time complexity of the testing procedure. The theory and practice of transparent tests with global and local symmetry are systematically presented in works [9,13,20,22], in which both the effectiveness of their applications and some limitations and disadvantages are discussed. Among the shortcomings, the poor diagnostic ability of these tests is highlighted, since the difference of some signature from its reference value (this is also the case in classical transparent tests) only allows obtaining the information of a faulty state of memory and does not indicate a fault. Thus, the known transparent technologies for testing memory devices are characterized by either a significant increase in time complexity or a decrease in diagnostic ability, and often both at the same time [7,9,13,20,22].

### 2.2. Analysis of the Effectiveness of Transparent Tests

It is important to note two features of transparent tests. First, transparent tests are built on the foundations of classical march tests, due to the fact that acceptable time complexity for such testing procedures is achieved only in the case of march tests [7,9,13,14]. Secondly, all existing tests, including transparent march tests, are always considered for memory containing *N* one-bit memory cells (*MC*), where typically $N=2^{m}$. In general, a march test consists of a finite number of march elements, often called test phases [14]. Each march element contains a symbol that determines the order of formation of the memory address sequence: ⇑ defines sequential enumeration of memory addresses in ascending order (in direct order), ⇓ determines sequential enumeration of addresses in descending order (in reverse order) and ⇕ signifies the selection of addresses in ascending or descending order [5,14]. In addition, the march element contains a sequence of read and write operations, enclosed in parentheses and separated by semicolons. Each operation is an element from the following set: r0 is the operation of reading the contents of the *MC* with the expected value of 0, r1 is the operation of reading the *MC* with the expected value of 1, w0 is the operation of writing 0 to the *MC* and w1 is the operation of writing 1 to the *MC*. One or more operations in the march element are used sequentially for the addressed memory cell. The transition to the next cell is carried out only after performing all operations with the current memory cell [5,14]. As an example of a march test, consider the March Y test: {⇕(w0);⇑(r0,w1,r1);⇓(r1,w0,r0);⇕(r0)}, which consists of four march elements and has complexity 8N. The first march element, ⇕(w0), is the initialization phase used to record the initial state of the memory. This phase is performed when the addresses change from the lowest addresses to the highest addresses, or conversely, by making all cells equal to zero. The second phase of the test, March Y ⇑(r0,w1,r1), determines the increasing order of addresses and consists of a read operation r0: when the expected value in an *MC* is 0, 1 is written in that cell. After that, the transition to the next memory cell is performed. The subsequent phases of this test are performed in the same way.

For a formal description of the faulty states of memory, mathematical models of their faults are used, reflecting the real physical defects of the memory [5,9,13,14,23,24]. Let us consider in more detail the dominating faults of the memory device (*MD*) using their generally accepted classification [14,24]. Faults affecting one memory cell include constant faults (stuck-at faults—SAF), when a faulty memory cell is permanently in a state of logical zero (SAF0) or a logical one (SAF1), regardless of the operations performed with the faulty *MC* and other cells. Transitions faults (TF) are characterized by the impossibility of transition of the state of a faulty *MC* from 0 to 1 (TF↑) or from 1 to 0 (TF↓) when performing the corresponding write operations [13,14].

Coupling faults (CF) are among the faults in which two memory cells are involved. When describing this fault, an influencing *MC* (aggressor cell) is noted, a change in the logical state of which affects the state of the dependent *MC* (victim cell). There are three types of mutual coupling faults. The dominant type of such faults are called direct-acting idempotent coupling faults (CF*id*), for which a change in the value of the influencing *MC* from zero to one ↑ or from one to zero ↓ leads to the forced setting of a certain logical value of 0 or 1 in the dependent *MC*. Eight CF*id* are distinguished:〈↑,0〉, 〈0,↑〉〈↑,1〉, 〈1,↑〉, 〈↓,0〉, 〈0,↓〉, 〈↓,1〉 and 〈1,↓〉 [14]. The location of the aggressor in relation to the victim is determined by the fault record; for example, in fault 〈0,↑〉, the address of the aggressor cell is less than the address of the victim cell, and in 〈↑,0〉, vice versa.

Pattern sensitive faults (PSFs) are viewed as a generalized class of the complex fault models. For such faults, the logical state or a change in the logical state of one *MC*, known as the base cell of the memory, may depend on the contents (0 or 1), or on the logical transitions from 1 to 0 or from 0 to 1, affecting the *MC* of the memory [13,14]. In the case of a pattern sensitive fault PSF*k*, in which *k* memory cells of the memory are involved, it is assumed in the limiting case that any k−1 out of the *N*MC of the memory can influence one base cell out of the remaining N−k+1MC [9]. In practice, restricted or neighborhood pattern sensitive faults (NPSF) are most often used. NPSFfaults involve a restriction which is imposed both on the number of *k* memory cells involved in the fault and on their physical locations. When modern memory testing devices are used, such faults typically adhere to the latest, most realistic model of pattern sensitive faults, for which a small number of k≤9 memory cells included in the NPSF*k* fault are considered, and their locations can be arbitrary. There are three classic NPSF*k* fault models: active (ANPSF*k*), passive (PNPSF*k*) and static (SNPSF*k*) [13,14]. Active or dynamic models include the NPSF*k* for which the base cell changes its content due to a change in the set stored in neighboring k−1 cells. Passive faults PNPSF*k* are those NPSF*k* for which the content of the base cell cannot be changed for a certain set of data in neighboring k−1 cells. Static SNPSF*k* are faults in which the content of the base cell is forced to one of two states, 0 or 1, due to a certain set in neighboring k−1 cells.

As an object of research, passive pattern sensitive faults (PNPSF*k*) are most often considered, where *k* denotes the number of arbitrary memory cells of MD with a capacity of *N* bits involved in a particular fault. Note that the results obtained for PNPSF*k* can be easily generalized to other classes of pattern sensitive faults, since PNPSF*k* is the memory fault model that is most difficult to detect and that covers other types of faults [13,14,25]. The number of all possible PNPSF*k* for MD with the *N* bits is determined according to expression (1) [13,26].

(1) QTN(PNPSFk)=k×2k×Nk.

The maximum possible number of $Q_{MAX}\left(PNPSFk\right)$ detected faults PNPSF*k* when using a single run march test is determined according to (2) [13,26].

(2) QMAX(PNPSFk)=(8×(k−2)+2×4)×Nk=8×(k−1)×Nk

Accordingly, single run march tests cannot exceed the maximum possible coverage of PNPSF*k* faults defined by expression (3) [13].

(3) $$FC_{MAX}\left(PNPSFk\right)=\frac{Q_{MAX}\left(PNPSFk\right)}{Q_{TN}\left(PNPSFk\right)}\times 100\%=\frac{k-1}{k\times 2^{k-3}}\times 100\%$$

Equation (3) shows the limited capabilities of classic march tests, in terms of detecting complex pattern sensitive faults PNPSF*k*. Using the previously described March Y test as an example, let us consider the PNPSF*k* fault detection efficiency of the original classic test and its transparent modifications. Table 1 shows the original March Y test and two of its transparent modifications [9,13,20].

In the descriptions of the tests presented in Table 1, value *a* takes an arbitrary meaning of 0 or 1, and $\bar{a}$ the inverse value with respect to *a*. The Nicolaidis transparent test consists of two parts, namely, the initial prediction test $\left\{⇑\left(ra,r\bar{a}\right);⇓\left(r\bar{a},ra\right);⇕\left(ra\right)\right\}$ which is necessary to obtain the reference signature $S_{F}$, and the base transparent test $\left\{⇑\left(ra,w\bar{a},r\bar{a}\right);⇓\left(r\bar{a},wa,ra\right);⇕\left(ra\right)\right\}$ [20]. During the base test implementation, a real $S_{R}$ signature is generated and then compared with the previously obtained reference $S_{F}$. Based on the comparison result, a decision is made on the presence or absence of faults in the memory. The symmetric transparent test does not require a preliminary calculation of the reference signature, as it is always standard, i.e., SF=000…0 [5,12]. In both transparent versions of the March Y test, the discrepancy between the real $S_{R}$ signature and its reference value $S_{F}$ indicates only a faulty memory state. Obtaining clarifying information about a fault requires additional time-consuming investigations [9,13,20].

Let us evaluate the effectiveness of the three versions of the March Y test shown in Table 1. First, consider the classical implementation of this test, for which 100% coverage was shown for the simplest faults such as SAF and TF [9,13,14,20]. For a fault of mutual influence such as CF*id*, only four of their eight types are detectable—namely, 〈↑,0〉, 〈↓,0〉, 〈1,↑〉 and 〈1,↓〉—giving only 50% of the fault coverage CF*id*. An even lower coverage of the March Y test is achieved in the class of complex pattern sensitive faults. For PNPSF*k* faults, only two of their types are detectable: 〈0,0,0,…,0,↑,1,1,1,…,1〉 and 〈0,0,0,...,0,↓,1,1,1,…,1〉. For example, for k=3, the March Y test detects 〈0,0,↑〉, 〈0,↑,1〉,〈↑,1,1〉, 〈↓,1,1〉, 〈0,↓,1〉 and 〈0,0↓〉, which is 6 out of $k\times 2^{k}=3\times 2^{3}=24$ faults in *k* fixed memory cells. In percentage terms, PSF3 coverage is only 25%. For arbitrary *k*, the fault coverage of PSF3 is calculated according to the expression (4).

(4) $$FC_{MarchY}\left(PNPSFk\right)=\frac{Q_{MarchY}\left(PNPSFk\right)}{Q_{TN}\left(PNPSFk\right)}\times 100\%=\frac{1}{2^{k-1}}\times 100\%$$

The efficiency of PNPSF*k* fault detection by transparent versions of the March Y test (see Table 1) is also quantitatively estimated using expression (4). However, unlike the classical implementation, transparent March Y will detect faults PNPSF*k* of the form $\langle a,a,a,\ldots ,a,↕,\bar{a},\bar{a},\bar{a},\ldots ,\bar{a}\rangle$ and $\langle \bar{a},\bar{a},\bar{a},\ldots ,\bar{a},↕,a,a,a,\ldots ,a\rangle$, where the symbol ↕ signifies inverting the current value *a* of the memory cell. A similar statement is also true for CF; however, in the first and second cases, the indicated faults may not be detected due to their mapping in the configuration of multiple errors, which can be masked when obtaining a real signature [27]. The effect of masking multiple errors by signature analysis also results in even single constant faults possibly not being detected. Therefore, the previously considered versions of transparent tests do not guarantee 100% detection of even the simplest memory faults [9,25].

## 3. Double Address Sequences

With a single application of march tests, including their transparent versions, the fault coverage of PNPSF*k*, along with any other faults, remains unchanged [13,25]. The only difference is the specific configurations of PNPSF*k* faults, which are detected (or not detected) by a transparent test with a fixed memory content and a given sequence of addresses [9,26]. Changing the contents of the memory during the operation of the computer system can significantly increase the fault coverage of PNPSF*k* with repeated use of transparent march tests [28]. However, as shown in [29], in the memory of modern computer systems, two characteristic components are distinguished: in one component, the content changes quite intensively, and in the second it remains practically unchanged. To improve the efficiency of multiple uses of march tests, a radical approach involves changing the sequence of addresses used in each of the subsequent iterations of the march test [25]. The range of uses of different address sequences is quite wide and was investigated in the frameworks of multiple tests [26,30,31]. The focus was on the choices of address sequences and their various modifications [13].

First, we will consider the general properties of address sequences and their modifications to implement transparent tests. Under the counter (counting) address sequence $A_{C}=A_{C}\left(0\right)A_{C}\left(1\right)A_{C}\left(2\right)\ldots A_{C}\left(N-2\right)A_{C}\left(N-1\right)$, where $A_{C}\left(j\right)\in \left\{0,1,2,\ldots ,N-1\right\}$, j∈{0,1,2,…,N−1} and $N=2^{m}$, we understand the sequence of addresses $A_{C}=c_{m-1}c_{m-2}\ldots c_{2}c_{1}c_{0}$, where $c_{i}\in \left\{0,1\right\}$ and i∈{0,1,2,…,m−1} are generated in accordance with the algorithm of the binary *m*-bit adding counter [13]. The starting address $A_{C}\left(0\right)$ of $A_{C}$ is the zero address $A_{C}\left(0\right)=000\ldots 0$, and the finish address is $A_{C}\left(N-1\right)=111\ldots 1$.

In the general case, an arbitrary address sequence $A=a_{m-1}a_{m-2}\ldots a_{2}a_{1}a_{0}$, where $a_{i}\in \left\{0,1\right\}$, i∈{0,1,2,…,m−1}, has the following properties [13]:

**Property** **1.** *The sequence of addresses $A=a_{m-1}a_{m-2}\ldots a_{2}a_{1}a_{0}$ consists of all possible $2^{m}$ addresses (binary combinations $a_{m-1}a_{m-2}\ldots a_{2}a_{1}a_{0}$) generated in arbitrary order, and each address is generated only once.*

**Property** **2.** *For any sequence of bits $a_{i}$ of addresses A, there are $2^{m-1}$ different binary combinations $a_{m-1}a_{m-2}\ldots a_{i+1}a_{i-1}\ldots a_{2}a_{1}a_{0}$ for $a_{i}=0$ and the same number of nonrepeating combinations $a_{m-1}a_{m-2}\ldots a_{i+1}a_{i-1}\ldots a_{2}a_{1}a_{0}$ for $a_{i}=1$.*

The last property can be generalized as the following statement:

**Statement** **1.** *An arbitrary collection of any m−1 bits $a_{m-1}a_{m-2}\ldots a_{i+1}a_{i-1}\ldots a_{2}a_{1}a_{0}$ out of m bits $a_{m-1}a_{m-2}\ldots a_{2}a_{1}a_{0}$ of the original address sequence A forms an address sequence in which each m−1 bit address is generated twice.*

As an example, Table 2 shows similar address sequences for the case of the original counter sequence $A_{C}=c_{3}c_{2}c_{1}c_{0}$ and the gray code sequence $A_{G}=g_{3}g_{2}g_{1}g_{0}$ for m=4.

Similar sequences with double repetitions of all addresses can be obtained in other ways, such as by permutations of both the addresses themselves and their bits of the original sequence obtained in accordance with Statement 1. In general, such sequences have to comply with the following definition:

**Definition** **1.** *The sequence of addresses $2A=a_{m-1}a_{m-2}\ldots a_{2}a_{1}a_{0}$ consists of all possible $2^{m}$ addresses (binary combinations $a_{m-1}a_{m-2}\ldots a_{2}a_{1}a_{0}$), each of which is generated twice, and their collection is formed in arbitrary order.*

Hereafter, such sequences with a period of $2^{m+1}$ will be referred to as double address sequences (2A), since each *m*-bit address is listed twice, as can be seen in Table 2.

An increasing sequence of such addresses will be denoted as 2⇑, and a decreasing sequence as 2⇓. For each address sequence in Table 2, their binary address values are shown, with their decimal equivalents presented in brackets. The form of the double address sequence depends both on the selected bits of the original address sequence *A* and on their permutations; accordingly, the total number of 2A sequences obtained from the original sequence *A* is equal to m!. Clearly, the number of such sequences for real values of *m* generated according to Statement 1 is high, as is the variety of their properties. As an example of the features of their properties, we note that in the sequence $2A=2A_{c0}=c_{3}c_{2}c_{1}$, all addresses are formed in time sequentially and in pairs, and in $2A=2A_{c3}=c_{2}c_{1}c_{0}$, the repeating addresses are maximally distant in time from each other (see Table 2).

The concept of transparent march tests utilizing double address sequences is based on the fact that when the contents of the memory cell are inverted twice, its value will remain the same. In accordance with this simplest property of the inversion operation, we construct a base element of a transparent march test based on a double address sequence 2A. As in classical transparent tests, the marching element must begin with the operation of reading ra of the contents of *a* of the current memory cell. This is necessary for unambiguous predicted actions with the current memory cell, which are based on knowledge of the meaning of its contents. The next operation is the operation of writing the inverse of the value just read from the cell content $\bar{a}$. This operation is followed by a read operation of the same current memory cell to check the correctness of the operation of inverting its contents. The next operation is to go to the following memory cell, which corresponds to the next address of the address sequence. The use of double address sequences 2A provides reinversion of each memory cell’s contents, resulting in its original state. Thus, the base element is written as follows:

(5) $$2⇑(ra,w\bar{a},ra).$$

Note that the use of a double address sequence (2A) in the base element means that each memory cell will sequentially perform two transitions from *a* to $\bar{a}$, and conversely, from $\bar{a}$ to *a*, thereby preserving its initial value. The base element performs the operation of reading both zero and one values from each memory cell and executing transitions from zero to one (↑) and from one to zero (↓) in it. The correctness of the transitions and the operations of reading zero and one values are ensured by the second operation of reading ra of the base element (5). To illustrate the implementation of the base element (5), let us consider its application for testing an MD containing N=8 cells with the initial content 00101110. The sequence of addresses $2A_{C}=2A_{c2}=c_{3}c_{1}c_{0}$ and $2AG=2A_{g2}=g_{3}g_{1}g_{0}$ is used as a double address sequence, provided in Table 2. The step-by-step change of the memory contents for both cases of double addressing is given in Table 3.

In each step of the implementation of the base element (5), only one memory cell changes its state to the opposite one. After completing all steps (5), each memory element will perform both transitions ↑ and ↓, and 0 and 1 will be read from each cell. As can be seen from Table 3, after the execution of the base element (5), the initial state of the memory remains unchanged.

The base element based on double address sequences allows the synthesis of two transparent march tests (6).

(6) $$\begin{array}{cc} March\_2A\_1: & \left\{⇕\left(ra\right);2⇑\left(ra,w\bar{a},ra\right);⇕\left(ra\right)\right\},\left(8N\right)\\ March\_2A\_2: & \left\{⇕\left(ra\right);2⇑\left(ra,w\bar{a},ra\right);2⇓\left(ra,w\bar{a},ra\right);⇕\left(ra\right)\right\},\left(14N\right) \end{array}$$

In both tests, the arbitrary order of addresses ⇕ for the first and last read operations must be the same. This is due to the fact that the first phase of the March_2A_1 and March_2A_2 tests is used to compress the initial state of the memory and obtain the reference $S_{F}$ signature, and the last phase is used to obtain the real value of the $S_{R}$ signature after the previous base elements have been executed. Regarding faults detected during the execution of base elements, their presence will be determined by the fulfillment of the inequality $S_{F}\neq S_{R}$.

## 4. Analysis of the Effectiveness of New Transparent Tests

Consider the fault coverage of new tests starting with the March_2A_1 test. Let us assume that in the case of the specified test the initial content of the memory is zero; that is, for all cells with a=0 and the base element $2⇕(ra,w\bar{a},ra)$ represented by two consecutive elements $⇑(ra,w\bar{a},ra)$ and $⇓(ra,w\bar{a},ra)$, we can conclude that the tests March_2A_1 and March Y are equivalent. Their equivalence lies both in the time complexity equal to 8N and in the ability to cover various types of faults. Let us sequentially consider the detection efficiency of March_2A_1 for the most significant types of memory faults.

As noted earlier, the base element (5) provides the activation and detection of all the simplest faults, such as SAF and TF. The write operation $w\bar{a}$ and double addressing 2A provide both the reading of zero and one from the current cell and the execution of two transitions of its state, creating a condition for activating these faults. Their detection is provided by the second read operation ra, the result of which is compared with the value obtained during the first read operation ra of the base element (5). In the absence of the indicated faults, these values should be opposite. If the values obtained during the implementation of the first and second operations of reading the base element are the same, this indicates the presence of faults, and in the case of SAF and TF, allows us to localize these faults. Thus, for the simplest faults, the proposed March_2A_1 test, in contrast to the known transparent tests, provides the maximum diagnostic ability.

Similarly, the maximum possible diagnostic ability for march tests by the March_2A_1 test is achieved for the case of complex pattern sensitive faults PNPSF*k*. Execution of the base element in the case of PNPSF*k* allows the identification of the address of the base cell, which cannot perform one of the transitions in this cell for specific content in neighboring cells. In addition, the March_2A_1 test achieves the same fault coverage for PNPSF*k* as the March Y test; in this case, only two of their types are detected, 〈u,u,u,…,u,↑,u,u,u,…,u〉 and 〈d,d,d,…,d,↓,d,d,d,…,d〉, where u,d∈{0,1}. The values of the contents of neighboring cells *u* and *d* depend both on the initial state of the MD and another type of sequence for addresses 2A. For example, for the case of an MD with eight cells and PNPSF3 faults in cells with addresses 1, 3 and 5, using the address sequence $2A_{c2}$, the base element (5) allows the detection of the following faults: 〈1,0,↑〉, 〈1,1,↓〉, 〈1,↑,1〉, 〈1,↓,0〉, 〈↑,1,1〉 and 〈↓,1,1〉 (see Table 3). Under the same conditions, changing the double address sequence $2A_{c2}$ to $2A_{g2}$ leads to the detection of another set of PNPSF3: 〈1,1,↑〉, 〈1,1,↓〉, 〈1,↑,0〉, 〈1,↓,0〉, 〈↑,1,1〉 and 〈↓,1,1〉 (see Table 3). Thus, a single application of the March_2A_1 test gives a fault coverage of 25% for PNPSF3, and its repeated use with variable 2A address sequences gives a fault coverage of 100% for arbitrary PNPSF*k*. The multiplicity of the test to achieve the maximum coverage completeness for a given *k* depends on many factors, and as in the case of classic march tests, requires additional analysis [7,8].

To detect the coupling faults, it is necessary to analyze the state of the victim’s cell after activating a specific fault, which is impossible within the framework of the base element (5). Therefore, these faults are detected when an even number of inversions of the contents of the victim’s cell by the base element changes by an odd number. Thus, the final state of the memory will differ from its initial state, which will lead to the fulfillment of the inequality $S_{F}\neq S_{R}$. Quantitatively, the fault coverage of the March_2A_1 test of such faults is equal to the fault coverage of the March Y test, as can be seen for CF*id* from the experimental data provided in Table 4.

The table shows that the total number of faults detected by the March_2A_1 test, regardless of the address sequence 2A, was always 50%. The data presented were obtained for a memory with a capacity of N=256 bits with a zero initial state. Address sequences $2A_{c0},\ldots ,2A_{c7}$, $2A_{c8}$ were formed from the counter sequence $A_{C}=c_{8}c_{7}c_{6}c_{5}c_{4}c_{3}c_{2}c_{1}c_{0}$ by removing the corresponding bit—for example, $2A_{c2}=c_{8}c_{7}c_{6}c_{5}c_{4}c_{3}c_{1}c_{0}$.

Analyzing the data given in Table 4 allowed us to conclude that the fault coverage of complex memory faults by the March_2A_1 test is independent from the address sequence used. Any address sequence corresponding to Definition 1 provides quantitatively the same fault coverage of complex memory faults. Regardless of the 2A address sequence used, the fault coverage for a given fault type remains unchanged. Changing the address sequence 2A affects the specific types of fault detected, as can be seen from the examples of CF*id* faults given in Table 4.

The March_2A_2 transparent march test differs from March_2A_1 by the presence of a second base element with reverse address order of 2A, which enhances its ability to detect complex faults while maintaining the effectiveness of March_2A_1 for simple faults. For example, the March_2A_1 test with the address sequence $2A_{c2}$ detects PNPSF3: 〈1,1,↑〉, 〈0,0,↓〉, 〈0,↑,0〉, 〈1,↓,1〉, 〈↑,1,0〉, and 〈↓,0,1〉 for storage cells 5, 6 and 7 (see Table 3). At the same time, the first base element of the March_2A_2 test detects the same PNPSF3, and the second base element additionally provides the detection of the following faults: 〈0,0,↑〉, 〈1,1,↓〉, 〈1,↑,1〉, 〈0,↓,0〉, 〈↑,0,1〉 and 〈↓,1,0〉. Thus, the number of PNPSF3 detected by the March_2A_2 test doubled compared to March_2A_1. Note that doubling the PNPSF*k* faults detected by the March_2A_2 test is achievable only for the case in which the first base element detects such faults as 〈u,u,u,…,u,↑,u,u,u,…,u〉 and 〈d,d,d,…,d,↓,d,d,d,…,d〉, for which the states of neighboring cells are different. Otherwise, the second base element will detect the same two faults 〈u,u,u,…,u,↑,u,u,u,…,u〉 and 〈d,d,d,…,d,↓,d,d,d,…,d〉, but in reverse order. For example, for the case of a memory device with eight cells and PNPSF3 faults in cells with addresses 1, 3 and 5, the first base element of the March_2A_2 test with the address sequence $2A_{c2}$ detects 〈↓,1,1〉 and 〈↑,1,1〉, among other faults (see Table 3). The second base element of the same March_2A_2 test also detects these faults, which ultimately does not lead to a twofold increase in PNPSF3 faults detected by this test. At the same time, as the value of *k* increases, the probability of the coincidence of the states of cells of neighbors in faults 〈u,u,u,…,u,↑,u,u,u,…,u〉 and 〈d,d,d,…,d,↓,d,d,d,…,d〉 noticeably decreases, resulting in a coverage completeness of PNPSF*k* close to 50%.

To ensure the fault coverage of the PNPSF*k* faults equal to 50%, it is necessary to select the appropriate address sequence. This statement is confirmed by the experimental data given in Table 5, which were obtained under the same conditions as the data shown in Table 4.

As can be seen from the above table, the fault coverage of CF*id* faults changed significantly, depending on the 2A address sequence used in the March_2A_2 test. Therefore, for the case in which $2A_{c0}=c_{8}c_{7}c_{6}c_{5}c_{4}c_{3}c_{2}c_{1}$, the fault coverage was only 50%; for $2A_{c8}=c_{7}c_{6}c_{5}c_{4}c_{3}c_{2}c_{1}$, the fault coverage reached a maximum value of 100%. Thus, in contrast to the single run test March_2A_1, the application of the March_2A_2 test required the choice of an address sequence to ensure high coverage of faults involving two or more memory cells.

## 5. March_2A_2 Test Requirements for Maximum Efficiency

Initially, we note that the standard one-run implementation of the test March_2A_1 does not depend on the specific type of address sequence 2A used. Any sequence of addresses 2A corresponding to Definition 1 will provide the same fault coverage for all types of memory faults discussed earlier. At the same time, for the March_2A_2 test, the type of addressing used by 2A is of fundamental importance; this is exemplified by the data provided in Table 5. For the most complex PNPSF*k* fault, the previous section showed that the March_2A_2 test, with an appropriate choice of 2A addressing, can double the fault coverage of PNPSF*k* compared with March_2A_1. When increasing the fault coverage of PNPSF*k* faults by factor of two, for fixed *k* cells, detection by the March_2A_2 test is achievable only for the case in which the first base element detects such faults as 〈u,u,u,…,u,↑,u,u,u,…,u〉 and 〈d,d,d,…,d,↓,d,d,d,…,d〉, for which the states of neighboring cells are different. Otherwise, the second base element will detect the same two faults, 〈u,u,u,…,u,↑,u,u,u,…,u〉 and 〈d,d,d,…,d,↓,d,d,d,…,d〉, but in reverse order, due to reverse addressing ⇓2A.

Two examples are provided in Table 6 and Table 7 to illustrate the importance of the address sequence 2A in terms of the fault coverage of the CF*id* and PNPSF*k* by one-run implementation of the March_2A_2 test. Both examples are given for the case of 8-bit memory with an all zero initial state. In the first case, $2A_{c0}=c_{3}c_{2}c_{1}$ was used as the address sequences, and in the second case, $2A_{c3}=c_{2}c_{1}c_{0}$ was used. Each table provides the step-by-step implementation of both phases of the March_2A_2 test (6).

As can be seen from Table 6, the second phase of the March_2A_2 test detects the same CF*id* and PNPSF*k* faults as the first phase of this test, but in reverse order. Moreover, this is true for any k≤N for memory of any size *N* and an arbitrary initial state. For the general case of an $2A_{c0}=c_{m}c_{m-1}\ldots c_{3}c_{2}c_{1}$ ($2A_{c0}=c_{3}c_{2}c_{1}$) address sequence, obtained from counting sequence $A_{C}$, only four CF*id*s, namely, 〈1,↑〉, 〈↑,1〉, 〈1,↓〉 and 〈↓,1〉, will be actually detectable by March_2A_2, as can be seen from Table 6 (see also Table 4 and Table 5). At the same time, the example shown in Table 6 indicates that only two PNPSF*k* faults, namely, 〈0,0,0…,0,↑,0,0,0,…,0〉 and 〈0,0,0,…,0,↓,0,0,0,…,0〉 are detectable.

A significantly different situation is illustrated by the second example shown in Table 7. Application of the address sequence $2A_{cm}=c_{m-1}c_{m-2}\ldots c_{2}c_{1}c_{0}$$\left(2A_{c3}=c_{2}c_{1}c_{0}\right)$ doubled the coverage of both CF*id* and PNPSF*k* faults. In this case, all eight types of CF*id*, 〈↑,0〉, 〈0,↑〉, 〈↑,1〉, 〈1,↑〉, 〈↓,0〉, 〈0,↓〉, 〈↓,1〉 and 〈1,↓〉; and four PNPSF*k* faults, 〈1,1,1…,1,↑,0,0,0,…,0〉, 〈0,0,0,…,0,↓,1,1,1,…,1〉, 〈1,1,1,…,1,↓,0,0,0,…,0〉 and 〈0,0,0,…,0,↑,1,1,1,…,1〉 were detected.

The two examples provided demonstrate the dependence of the effectiveness of the March_2A_2 test on the selected address sequences 2A. This is true for any arbitrary address sequence satisfying Definition 1, and not only for 2A obtained from the counter sequence AC.

Based on these examples of the March_2A_2 test implementation for different 2A address sequences, we can formulate the conditions for achieving the maximum coverage of faults CF*id* and PNPSF*k* with a one-run March_2A_2 test.

Regarding CF*id* faults, 100% fault coverage will be achieved if the following four states are formed for each N−1 remaining memory cell of the memory, when the aggressor cell makes the transitions (7).

(7) $$\begin{array}{ccccccccccc} b & b & b & \ldots & b & ↑ & \bar{b} & \bar{b} & \bar{b} & \ldots & \bar{b}\\ \bar{b} & \bar{b} & \bar{b} & \ldots & \bar{b} & ↓ & b & b & b & \ldots & b\\ b & b & b & \ldots & b & ↓ & \bar{b} & \bar{b} & \bar{b} & \ldots & \bar{b}\\ \bar{b} & \bar{b} & \bar{b} & \ldots & \bar{b} & ↑ & b & b & b & \ldots & b \end{array}$$

Indeed, for an arbitrary state b∈{0,1} in each of presumed victim cells, the fulfillment of condition (7) will lead to the detection of all eight CF*id* faults: 〈b,↑〉, $\langle \bar{b},↑\rangle$, 〈b,↓〉, $\langle \bar{b},↓\rangle$, 〈↑,b〉, $\langle ↑,\bar{b}\rangle$, 〈↓,b〉 and $\langle ↓,\bar{b}\rangle$.

Initially, it should be noted that the maximum number of PNPSF*k* faults detected by any single-run march test, including the March_2A_1 and March_2A_2 tests, is equal to the number of write operations of inverse values used in the test. This means that for any *k* fixed memory cells and for each aggressor cell, the March_2A_1 test detects two faults, and the March_2A_2 test detects a maximum of four PNPSF*k* faults.

As noted earlier, the single test March_2A_1 detects two PNPSF*k* faults, 〈u,u,u,…,u,↑,u,u,u,…,u〉 and 〈d,d,d,…,d,↓,d,d,d,…,d〉, where u,d∈{0,1} for any *k*, including k=N, and the following inequality:

(8) u,u,u,…,u≠d,d,d,…,d

For the states of the k−1 neighboring cells, excluding the base cell for these two faults is a necessary and sufficient condition to double the fault coverage of PNPSF*k* by the March_2A_2 for a given *k*. However, the fulfillment of condition (8), which ensures the detection of four PNPSF*k* faults for a given number *k* of fixed memory cells, does not guarantee the detection of PNPSF*k* faults for smaller values of *k*. For example, for two faults 〈1,↑,1,1〉 and 〈0,↓,1,1〉 for 2, 3, 4 and 5 memory cells, as shown in Table 3, the conditions of (8) satisfy 1,1,1≠0,1,1, and two additional PNPSF4〈1,↓,1,1〉 and 〈0,↑,1,1〉 are detected by the March_2A_2 test. At the same time, for k=3 and memory cells with addresses 2, 3 and 4, only two PNPSF3 faults are detected by March_2A_2, namely, 〈↑,1,1〉 and 〈↓,1,1〉, due to the nonfulfillment of condition (8). To avoid such situations, condition (8) must be extended to the case in which $d,d,d,\ldots ,d=\bar{u},\bar{u},\bar{u},\ldots ,\bar{u}$.

The above analysis allowed us to formulate a generalized requirement for the March_2A_2 test, and accordingly, for the address sequence 2A used to implement it.

To achieve the maximum fault detection ability by the March_2A_2 test, for any arbitrary cell acting as an aggressor (in a case CF*id*) or as a base cell (in a case PNPSF*k*), the following condition must be met for all remaining N−1 cells:

(9) $$\begin{array}{cccccccccccc} b & b & b & \ldots & b & ↑ & \bar{b} & \bar{b} & \bar{b} & \ldots & \bar{b} & \\ \bar{b} & \bar{b} & \bar{b} & \ldots & \bar{b} & ↓ & b & b & b & \ldots & b & . \end{array}$$

In this case, the symbols ↑ and ↓ denote an arbitrary cell, and the random b∈{0,1} states of the remaining cells are changed in an orderly manner to the opposite values $\bar{b}$.

## 6. Investigation of the Properties of Double Address Sequences 2A

The sequence of addresses $2A=a_{m-1}a_{m-2}\ldots a_{2}a_{1}a_{0}$ consists of all possible $2^{m}$ addresses $A\left(0\right)A\left(1\right)A\left(2\right)\ldots A\left(2^{m}-2\right)A\left(2^{m}-1\right)$, where $A\left(j\right)\in \left\{0,1,2,\ldots ,2^{m}-1\right\}$, $j\in \{0,1,2,\ldots ,2^{m}-1\}$ are binary combinations $a_{m-1}a_{m-2}\ldots a_{2}a_{1}a_{0}$, each of which is generated twice. This means that in an arbitrary sequence 2A, two addresses A(j) are generated at a certain distance from each other, which is determined by the algorithm for generating 2A. For example, for the address sequence $2A_{C}=c_{2}c_{1}c_{0}$, addresses A(1)=001 are generated at a distance of 8 from each other, and the same two addresses in the sequence $2A_{G}=g_{2}g_{1}g_{0}$ are generated at a distance of 13 (see Table 3). Thus, each address A(j) of the 2A sequence has a fixed distance between its repeated generation.

For the general case, an arbitrary address A(j) has two values of the distance metric AD(A(j),2A), namely, *r* and $2^{m+1}-r$, which depend on the starting A(0) address of the 2A sequence. This follows from the properties of cyclic sequences, and can be explained as follows. Given that the period of the sequence of addresses 2A is equal to $2^{m+1}$, and the distance between the first and second addresses A(j) is equal to *r*, the distance between the second and the first addresses A(j) will be equal to $2^{m+1}-r$. In the previous example, the starting address for both $2A_{C}=c_{2}c_{1}c_{0}$ and $2A_{G}=g_{2}g_{1}g_{0}$ sequences was an all-zero address A(0)=000. Changing the starting address to A(3)=011 would cause $AD(A\left(1\right),2A_{G})$ to change its value r=13 by $2^{m+1}-r=24–13=3$, and $AD(A\left(1\right),2A_{C})$ would remain unchanged at 8.

A more general characteristic describing the sequence 2A determines the average distance of addresses and is calculated according to the following expression:

$$SD\left(2A\right)=\frac{1}{2^{m}}\sum_{j=0}^{2^{m}-1}AD(A(j),2A).$$

Specifically, we will assume that both metrics are calculated at a zero starting address A(0), understanding that they both depend on it; AD(A(j),2A) is the distance between the first A(j) and second, and not vice versa. For example, for the sequence $2A_{G}=g_{2}g_{1}g_{0}$, the average address distance SD(2A) is calculated as follows: $SD\left(2A_{G}\right)=\left(15+13+9+11+1+3+7+5\right)/8=8$ (see Table 3).

Furthermore, we use the metric of the average distance ASD(2A) between the same addresses A(j) of sequence 2A, which is invariant with respect to the starting address. This metric is calculated according to expression (10).

(10) $$ASD\left(2,A\right)=\frac{1}{2^{m}}\sum_{j=0}^{2^{m}-1}MIN[AD\left(A\left(j\right),2A\right),\left(2^{m-1}-AD\left(A\left(j\right),2A\right)\right)]$$

Then, for the same example $2A_{G}=g_{2}g_{1}g_{0}$, the average distance between the same addresses $2A_{G}$ is calculated according to (10) as 4, and this value is repeated for any initial values of the address.

Now consider the main properties of two metrics, AD(A(j),2A) and ASD(2A).

1. The minimum value of the metric AD(A(j),2A) does not depend on the starting address of the sequence 2A, so for any starting address, it takes one out of two values, *r* or $2^{m+1}-r$. Similarly, the value of the metric ASD(2A) does not depend on the starting address of the sequence 2A.
2. For any sequence 2A with period equal to $2^{m+1}$ satisfying Definition 1, the metrics AD(A(j),2A) and ASD(2A) take the following values:
  (11) $$\begin{array}{c} 1\leq AD\left(A\left(j\right),2A\right)\leq 2^{m+1}-1\\ 1\leq ASD\left(2A\right)\leq 2^{m} \end{array}$$
3. The maximum value of the metric ASD(2A) is achieved when $AD\left(A\left(j\right),2A\right)=2^{m}$ for all $j\in \{0,1,2,\ldots ,2^{m}-1\}$, as described by expression (10).

The metrics AD(A(j),2A) and ASD(2A) for the sequence 2A allow us to formulate condition (9) for the maximum efficiency of the March_2A_2 test in terms of these characteristics. Indeed, only in the case in which for any memory cell with the address A(j), the distance AD(A(j),2A) will be equal to $2^{m}$, in the remaining memory cells, the inverse states given in (9) will be formed. Recall that condition (9) was formulated for all memory cells, the number of which, *N*, equals $2^{m}$.

Thus, the condition for the maximum effectiveness of the March_2A_2 test can be formulated as the following statement:

**Statement** **2.** *The maximum efficiency of the* *March_2A_2* *test, characterized by 100% coverage of* *SAF* *,* *TF* *and* *CF* id *faults, and fault coverage equal to $1/2^{k-2}\times 100\%$ for* *PNPSF* k *, is achieved when using the address sequence 2A, for which $ASD\left(2A\right)=2^{m}$.*

The value $1/2^{k-2}\times 100\%$ of the coverage of PNPSF*k* faults follows from the fact that the March_2A_2 test detects four types of similar faults determined by the condition (9) fulfilled by their detection.

It should be noted that the address sequence $2A_{Cm}=c_{m-1}c_{m-2}\ldots c_{2}c_{1}c_{0}$, $\left(2A_{c3}=c_{2}c_{1}c_{0}\right)$ given in Table 7 for m=3 satisfies Statement 1 and provides the maximum covering ability of the March_2A_2 test.

## 7. Conclusions

This article presented a new approach for constructing transparent memory tests based on the use of address sequences with repeated addresses. The primary innovation in this work is the base element (5) based on the use of double address sequences 2A. The use of this element for constructing tests March_2A_1 and March_2A_2 significantly reduces the complexity of transparent tests in comparison with the known approaches, and in particular, with the classic transparent Nicolaidis tests. The second clear advantage of the new tests is their diagnostic ability, which is comparable to the diagnostic ability of march tests, particularly for simple faults such as SAF and TF. The third definite advantage is the preservation of the covering ability of the March_2A_1 test in relation to the test March Y due to the elimination of the masking effect of errors caused by memory faults, since any memory fault is transformed by both tests if errors of a multiplicity do not exceed two. Newly introduced double address sequence metrics allow the selection of address sequences to maximize the effectiveness of the March_2A_2 test. These metrics make it possible to further synthesize the repeated use of the developed tests March_2A_1 and March_2A_2 to achieve their maximum efficiency with the minimum number of runs.

## Figures and Tables

**Table 1:** Three versions of the March Y test implementation.

Description	Test	Complexity
March Y test	{⇕(w0);⇑(r0,w1,r1);⇓(r1,w0,r0);⇕(r0)}	8*N*
Transparent Nicolaidis test	$\left\{⇑\left(ra,r\bar{a}\right);⇓\left(r\bar{a},ra\right);⇕\left(ra\right)\right\}$ $\left\{⇑\;\left(ra,w\bar{a},r\bar{a}\right);⇓\left(r\bar{a},wa,ra\right);⇕\left(ra\right)\right\}$	12*N*
Symmetric test	$\left\{⇓\left(r\bar{a}\right);⇑\left(r\bar{a},ra,w\bar{a},r\bar{a}\right);⇓\;\left(ra,r\bar{a},wa,ra\right);⇕\left(ra\right)\right\}$	10*N*

**Table 2:** Address sequences $A_{C}$ and $A_{G}$, and their $2A_{C}$ and $2A_{G}$ modifications.

$A_{C}=c_{3}c_{2}c_{1}c_{0}$	$2A_{C}=c_{3}c_{2}c_{1}$	$2A_{C}=c_{3}c_{2}c_{0}$	$2A_{C}=c_{3}c_{1}c_{0}$	$2A_{C}=c_{2}c_{1}c_{0}$	$A_{G}=g_{3}g_{2}g_{1}g_{0}$	$2A_{G}=g_{3}g_{2}g_{1}$	$2A_{G}=g_{3}g_{2}g_{0}$	$2A_{G}=g_{3}g_{1}g_{0}$	$2A_{G}=g_{2}g_{1}g_{0}$
0000 (0)	000 (0)	000 (0)	000 (0)	000 (0)	0000 (0)	000 (0)	000 (0)	000 (0)	000 (0)
0001 (1)	000 (0)	001 (1)	001 (1)	001 (1)	0001 (1)	000 (0)	001 (1)	001 (1)	001 (1)
0010 (2)	001 (1)	000 (0)	010 (2)	010 (2)	0011 (3)	001 (1)	001 (1)	011 (3)	011 (3)
0011 (3)	001 (1)	001 (1)	011 (3)	011 (3)	0010 (2)	001 (1)	000 (0)	010 (2)	010 (2)
0100 (4)	010 (2)	010 (2)	000 (0)	100 (4)	0110 (6)	011 (3)	010 (2)	010 (2)	110 (6)
0101 (5)	010 (2)	011 (3)	001 (1)	101 (5)	0111 (7)	011 (3)	011 (3)	011 (3)	111 (7)
0110 (6)	011 (3)	010 (2)	010 (2)	110 (6)	0101 (5)	010 (2)	011 (3)	001 (1)	101 (5)
0111 (7)	011 (3)	011 (3)	011 (3)	111 (7)	0100 (4)	010 (2)	010 (2)	000 (0)	100 (4)
1000 (8)	100 (4)	100 (4)	100 (4)	000 (0)	1100 (12)	110 (6)	110 (6)	100 (4)	100 (4)
1001 (9)	100 (4)	101 (5)	101 (5)	001 (1)	1101 (13)	110 (6)	111 (7)	101 (5)	101 (5)
1010 (10)	101 (5)	100 (4)	110 (6)	010 (2)	1111 (15)	111 (7)	111 (7)	111 (7)	111 (7)
1011 (11)	101 (5)	101 (5)	111 (7)	011 (3)	1110 (14)	111 (7)	110 (6)	110 (6)	110 (6)
1100 (12)	110 (6)	110 (6)	100 (4)	100 (4)	1010 (10)	101 (5)	100 (4)	110 (6)	010 (2)
1101 (13)	110 (6)	111 (7)	101 (5)	101 (5)	1011 (11)	101 (5)	101 (5)	111 (7)	011 (3)
1110 (14)	111 (7)	110 (6)	110 (6)	110 (6)	1001 (9)	100 (4)	101 (5)	101 (5)	001 (1)
1111 (15)	111 (7)	111 (7)	111 (7)	111 (7)	1000 (8)	100 (4)	100 (4)	100 (4)	000 (0)

**Table 3:** The procedure for implementing the base element (5) of a nondestructive test for two types of addressing.

MD	Addr.	7	6	5	4	3	2	1	0	MD	Address	7	6	5	4	3	2	1	0
	Content	0	0	1	0	1	1	1	0		Content	0	0	1	0	1	1	1	0
$2A_{c2}$	000 (0)	0	0	1	0	1	1	1	↑	$2A_{g2}$	000 (0)	0	0	1	0	1	1	1	↑
	001 (1)	0	0	1	0	1	1	↓	1		001 (1)	0	0	1	0	1	1	↓	1
	010 (2)	0	0	1	0	1	↓	0	1		011 (3)	0	0	1	0	↓	1	0	1
	011 (3)	0	0	1	0	↓	0	0	1		010 (2)	0	0	1	0	0	↓	0	1
	000 (0)	0	0	1	0	0	0	0	↓		010 (2)	0	0	1	0	0	↑	0	1
	001 (1)	0	0	1	0	0	0	↑	0		011 (3)	0	0	1	0	↑	1	0	1
	010 (2)	0	0	1	0	0	↑	1	0		001 (1)	0	0	1	0	1	1	↑	1
	011 (3)	0	0	1	0	↑	1	1	0		000 (0)	0	0	1	0	1	1	1	↓
	100 (4)	0	0	1	↑	1	1	1	0		100 (4)	0	0	1	↑	1	1	1	0
	101 (5)	0	0	↓	1	1	1	1	0		101 (5)	0	0	↓	1	1	1	1	0
	110 (6)	0	↑	0	1	1	1	1	0		111 (7)	↑	0	0	1	1	1	1	0
	111 (7)	↑	1	0	1	1	1	1	0		110 (6)	1	↑	0	1	1	1	1	0
	100 (4)	1	1	0	↓	1	1	1	0		110 (6)	1	↓	0	1	1	1	1	0
	101 (5)	1	1	↑	0	1	1	1	0		111 (7)	↓	0	0	1	1	1	1	0
	110 (6)	1	↓	1	0	1	1	1	0		101 (5)	0	0	↑	1	1	1	1	0
	111 (7)	↓	0	1	0	1	1	1	0		100 (4)	0	0	1	↓	1	1	1	0

**Table 4:** Fault coverage of CF*id* faults by March_2A_1 test in percent (%).

CFid	$2A_{c0}$	$2A_{c1}$	$2A_{c2}$	$2A_{c3}$	$2A_{c4}$	$2A_{c5}$	$2A_{c6}$	$2A_{c7}$	$2A_{c8}$
〈0,↑〉	0.00	0.00	0.00	0.00	0.00	0.00	0.00	0.00	0.00
〈1,↑〉	100.00	100.00	100.00	100.00	100.00	100.00	100.00	100.00	100.00
〈0,↓〉	0.00	0.39	1.18	2.75	5.88	12.16	24.71	49.80	100.00
〈1,↓〉	100.00	99.61	98.82	97.25	94.12	87.84	75.29	50.20	0.00
〈↑,0〉	0.00	0.39	1.18	2.75	5.88	12.16	24.71	49.80	100.00
〈↑,1〉	100.00	99.61	98.82	97.25	94.12	87.84	75.29	50.20	0.00
〈↓,0〉	0.00	0.00	0.00	0.00	0.00	0.00	0.00	0.00	0.00
〈↓,1〉	100.00	100.00	100.00	100.00	100.00	100.00	100.00	100.00	100.00
Total	50.00	50.00	50.00	50.00	50.00	50.00	50.00	50.00	50.00

**Table 5:** Fault coverage of CF*id* faults by March_2A_2 test in percent (%).

CF *id*	$2A_{c0}$	$2A_{c1}$	$2A_{c2}$	$2A_{c3}$	$2A_{c4}$	$2A_{c5}$	$2A_{c6}$	$2A_{c7}$	$2A_{c8}$
〈0,↑〉	0.00	0.39	1.18	2.75	5.88	12.16	24.71	49.80	100.00
〈1,↑〉	100.00	100.00	100.00	100.00	100.00	100.00	100.00	100.00	100.00
〈0,↓〉	0.00	0.39	1.18	2.75	5.88	12.16	24.71	49.80	100.00
〈1,↓〉	100.00	100.00	100.00	100.00	100.00	100.00	100.00	100.00	100.00
〈↑,0〉	0.00	0.39	1.18	2.75	5.88	12.16	24.71	49.80	100.00
〈↑,1〉	100.00	100.00	100.00	100.00	100.00	100.00	100.00	100.00	100.00
〈↓,0〉	0.00	0.39	1.18	2.75	5.88	12.16	24.71	49.80	100.00
〈↓,1〉	100.00	100.00	100.00	100.00	100.00	100.00	100.00	100.00	100.00
Total	50.00	50.20	50.59	51.37	52.94	56.08	62.35	74.99	100.00

**Table 6:** One-run implementation of the March_2A_2 test with $2A_{c0}$ addressing.

MD	Addr.	7	6	5	4	3	2	1	0	MD	Addr.	7	6	5	4	3	2	1	0
	Content	0	0	0	0	0	0	0	0		Content	0	0	0	0	0	0	0	0
First	000 (0)	0	0	0	0	0	0	0	↑	Second	111 (7)	↑	0	0	0	0	0	0	0
phase	000 (0)	0	0	0	0	0	0	0	↓	phase	111 (7)	↓	0	0	0	0	0	0	0
$⇑2A_{c0}$	001 (1)	0	0	0	0	0	0	↑	0	$⇓2A_{c0}$	110 (6)	0	↑	0	0	0	0	0	0
	001 (1)	0	0	0	0	0	0	↓	0		110 (6)	0	↓	0	0	0	0	0	0
	010 (2)	0	0	0	0	0	↑	0	0		101 (5)	0	0	↑	0	0	0	0	0
	010 (2)	0	0	0	0	0	↓	0	0		101 (5)	0	0	↓	0	0	0	0	0
	011 (3)	0	0	0	0	↑	0	0	0		100 (4)	0	0	0	↑	0	0	0	0
	011 (3)	0	0	0	0	↓	0	0	0		100 (4)	0	0	0	↓	0	0	0	0
	100 (4)	0	0	0	↑	0	0	0	0		011 (3)	0	0	0	0	↑	0	0	0
	100 (4)	0	0	0	↓	0	0	0	0		011 (3)	0	0	0	0	↓	0	0	0
	101 (5)	0	0	↑	0	0	0	0	0		010 (2)	0	0	0	0	0	↑	0	0
	101 (5)	0	0	↓	0	0	0	0	0		010 (2)	0	0	0	0	0	↓	0	0
	110 (6)	0	↑	0	0	0	0	0	0		001 (1)	0	0	0	0	0	0	↑	0
	110 (6)	0	↓	0	0	0	0	0	0		001 (1)	0	0	0	0	0	0	↓	0
	111 (7)	↑	0	0	0	0	0	0	0		000 (0)	0	0	0	0	0	0	0	↑
	111 (7)	↓	0	0	0	0	0	0	0		000 (0)	0	0	0	0	0	0	0	↓

**Table 7:** One-run implementation of the March_2A_2 test with $2A_{c3}$ addressing.

MD	Addr.	7	6	5	4	3	2	1	0	MD	Addr.	7	6	5	4	3	2	1	0
	Content	0	0	0	0	0	0	0	0		Content	0	0	0	0	0	0	0	0
First	000 (0)	0	0	0	0	0	0	0	↑	Second	111 (7)	↑	0	0	0	0	0	0	0
phase	001 (1)	0	0	0	0	0	0	↑	1	phase	110 (6)	1	↑	0	0	0	0	0	0
$⇑2A_{c3}$	010 (2)	0	0	0	0	0	↑	1	1	$⇓2A_{c3}$	101 (5)	1	1	↑	0	0	0	0	0
	011 (3)	0	0	0	0	↑	1	1	1		100 (4)	1	1	1	↑	0	0	0	0
	100 (4)	0	0	0	↑	1	1	1	1		011 (3)	1	1	1	1	↑	0	0	0
	101 (5)	0	0	↑	1	1	1	1	1		010 (2)	1	1	1	1	1	↑	0	0
	110 (6)	0	↑	1	1	1	1	1	1		001 (1)	1	1	1	1	1	1	↑	0
	111 (7)	↑	1	1	1	1	1	1	1		000 (0)	1	1	1	1	1	1	1	↑
	000 (0)	1	1	1	1	1	1	1	↓		111 (7)	↓	1	1	1	1	1	1	1
	001 (1)	1	1	1	1	1	1	↓	0		110 (6)	0	↓	1	1	1	1	1	1
	010 (2)	1	1	1	1	1	↓	0	0		101 (5)	0	0	↓	1	1	1	1	1
	011 (3)	1	1	1	1	↓	0	0	0		100 (4)	0	0	0	↓	1	1	1	1
	100 (4)	1	1	1	↓	0	0	0	0		011 (3)	0	0	0	0	↓	1	1	1
	101 (5)	1	1	↓	0	0	0	0	0		010 (2)	0	0	0	0	0	↓	1	1
	110 (6)	1	↓	0	0	0	0	0	0		001 (1)	0	0	0	0	0	0	↓	1
	111 (7)	↓	0	0	0	0	0	0	0		000 (0)	0	0	0	0	0	0	0	↓
